# On the Remedial Agency of the Air Pump

**Published:** 1836-10-01

**Authors:** 


### On the Remedial Agency of the Air-pump. By *Sir James Murray*, M.D.

We have often thought that dry-cupping was not so much employed as it ought to be. If atmospheric pressure could be taken off one part of the body without adding to the pressure on some other part, there can be no doubt the air-pump would be a most powerful remedial agent. But this cannot be done, though Sir James Murray seems to have obviated the difficulty of the attempt, as far as ingenuity could do so.

"The consideration of these difficulties led me to contrive a metallic case for embracing the whole hand, with an adaption to render it air-tight, either above or below the elbow, a pipe to connect it with the exhausting syringe, and another to attach with a mercurial gauge, which should regulate the degree of vacuum. When the rarefaction is properly effected, it can be continued for any length of time which may be considered requisite for the protection of the patient from the effects of poisoned wounds, as well as the bites of rabid animals, and similar sources of deleterious matter." 35.

The apparatus for taking off atmospheric pressure from the general surface of the body—at least from the major part of it—is thus described.

"For the purpose of insulating a patient's body from the external air, a small oval bath, of tin, zinc, or copper, is employed; its lip is furnished with a groove to contain luting, for connecting the lid or cover.

In this lid is an aperture to pass over the patient's head, and around this opening is fitted a margin of air-tight cloth, which applies itself so as to embrace the top of the chest and back of the neck. The patient sits upon a seat, easily varied to a regular height, and his head is unconfined, the body and limbs only being inclosed. When the bath is thus adjusted, it is to be partially rarefied, either by the condensation of a little hot air or steam, or by a few strokes of the suction-pump.

When the air of the bath is exhausted, to the extent of two inches, upon the mercurial gauge, it indicates the removal of *one hundred and forty-four pounds* from every square foot of the patient's surface, or one pound from every square inch.

Rarefaction to this degree is the utmost at any time required, and does not injuriously interfere with those great hydraulic laws, which modify either the general or pulmonary circulation. But, on the other hand, when the principle is applied *topically* to a part of the trunk, or to the limbs, as no interference with respiration can then occur, *two or three pounds* of pressure may be drawn from every square inch, but there is seldom occasion to proceed to that extent. To draw away even a fifteenth part of the atmospheric gravity will, I think, be found a therapeutic agent, abundantly powerful for any useful purpose.

It was my intention to give drawings of the various adaptations, but as it is obvious that any misconception might lead to injurious results, I have thought it safer to direct an able artist, Mr. Hodges of Westmorland-street, to make the apparatus, supply proper directions, and send a careful person conversant with its mechanism to apply it. He has also arranged a similar facility, to be afforded by Mr. Hart, 25, Hunter-street, Hanover-square, London, and any other place that may require it.

I have further requested him to communicate to any medical gentleman, the most free and explicit explanations respecting the mechanism and applications of the general or local baths for Rarefaction." 43.

The author has employed local rarefaction in several cases, and suggests it and general rarefaction in many complaints. We shall glance at some of these.

*Case* 1. A man had laboured for several months under excruciating pain in the right side of the head, for which various remedies were given without relief. One leg and thigh were enclosed in the exhausting air-bath for an hour and a half, by which the limb was enormously swelled, and the capillaries highly injected. The head-ache was relieved.

*Case* 2. A painter had, during seven years, complained of pains and weakness of the arms and wrists, which incapacitated him for his work. The right arm was enclosed in a tin case, and suction effected. The fingers became gradually hot, then the wrist, and lastly the elbow. When the exhaustion had continued half an hour, he felt the perspiration copious, the fingers as if pulled out and expanded, and the hand enlarged, so as to fill the case. In a few days he was able to work.

Rarefaction of the air, when a limb is severely burnt, brings back, our author remarks, a supply of blood and nervous energy, when the circulation becomes languid, and sphacelation is threatened. In partial paralysis, he thinks, we may give (where there is coldness and defective nervous energy) an impulse to the nerves through the process of suction. In respect to the limbs it is a species of dry-cupping, for new purposes and on a new scale.

"The trunk may be compared to a hollow tube, or case, filled by our vital organs: abstracting a part of the surrounding compression from the exterior of the body, permits its easier expansion, dilates its interior, and allows the great viscera to unload themselves of the surcharge of fluids, frequently detained in their vessels and spongy membranes.

When we reflect upon the laws of hydraulics, and consider that the brain and its blood are incompressible in their air-tight case, it will at once be admitted, how important it is in practice, to adopt means in addition to those usually had recourse to, capable of abating an untoward impetus of the circulation to the brain." 31.

With this extract we must close our notice of Sir James's pamphlet. We think the subject deserves further investigation.

---

